# Stiffened fibre-like microenvironment based on patterned equidistant micropillars directs chondrocyte hypertrophy

**DOI:** 10.1016/j.mtbio.2023.100682

**Published:** 2023-05-27

**Authors:** Mengmeng Duan, Shuang Xia, Yang Liu, Xiaohua Pu, Yukun Chen, Yilin Zhou, Minglei Huang, Caixia Pi, Demao Zhang, Jing Xie

**Affiliations:** a State Key Laboratory of Oral Diseases, West China Hospital of Stomatology, Sichuan University, Chengdu, China; b State Key Laboratory of Polymer Materials Engineering, Polymer Research Institute of Sichuan University, Chengdu, 610065, China; c National Clinical Research Center for Oral Diseases, West China Hospital of Stomatology, Sichuan University, Chengdu, 610041, China; d Institute of Biomedical Engineering, West China School of Basic Medical Sciences & Forensic Medicine, Sichuan University, Chengdu, China

**Keywords:** Micropillar substrate, ECM stiffness, Chondrocyte hypertrophy, Mechanoreception, Mechanotransduction

## Abstract

Articular cartilage, composed of collagen type II as a major extracellular matrix and chondrocyte as a unique cell type, is a specialized connective tissue without blood vessels, lymphatic vessels and nerves. This distinctive characteristic of articular cartilage determines its very limited ability to repair when damaged. It is well known that physical microenvironmental signals regulate many cell behaviors such as cell morphology, adhesion, proliferation and cell communication even determine chondrocyte fate. Interestingly, with increasing age or progression of joint diseases such as osteoarthritis (OA), the major collagen fibrils in the extracellular matrix of articular cartilage become larger in diameter, leading to stiffening of articular tissue and reducing its resistance to external tension, which in turn aggravates joint damage or progression of joint diseases. Therefore, designing a physical microenvironment closer to the real tissue and thus obtaining data closer to the real cellular behaviour, and then revealing the biological mechanisms of chondrocytes in pathological states is of crucial importance for the treatment of OA disease. Here we fabricated micropillar substrates with the same topology but different stiffnesses to mimic the matrix stiffening that occurs in the transition from normal to diseased cartilage. It was first found that chondrocytes responded to stiffened micropillar substrates by showing a larger cell spreading area, a stronger enhancement of cytoskeleton rearrangement and more stability of focal adhesion plaques. The activation of Erk/MAPK signalling in chondrocytes was detected in response to the stiffened micropillar substrate. Interestingly, a larger nuclear spreading area of chondrocytes at the interface layer between the cells and top surfaces of micropillars was observed in response to the stiffened micropillar substrate. Finally, it was found that the stiffened micropillar substrate promoted chondrocyte hypertrophy. Taken together, these results revealed the cell responses of chondrocytes in terms of cell morphology, cytoskeleton, focal adhesion, nuclei and cell hypertrophy, and may be beneficial for understanding the cellular functional changes affected by the matrix stiffening that occurs during the transition from a normal state to a state of osteoarthritis.

## Graphical abstract

**Figure:**
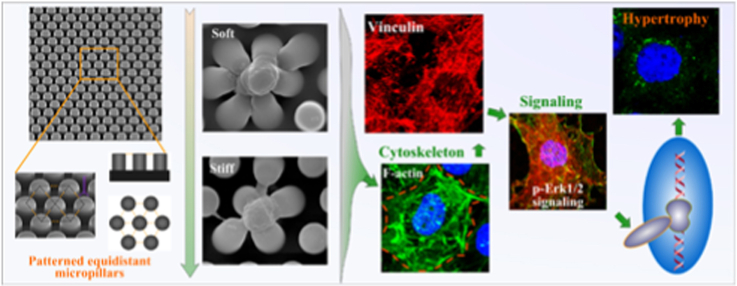

## Statement of significance

It is a major challenge to design a physical microenvironment that mimics that of real tissue, thus allowing data on cell behaviour that more closely represents real cell behaviour. The current study provides a kind of patterned equidistant micropillar substrate based on PDMS that mimics the collagen fibre-like network of the cartilage matrix. By changing the curing agent, we obtained soft and stiff micropillar substrates with the same topology to mimic the matrix stiffening that occurs in cartilage during the transition from a normal state to a state of osteoarthritis. We performed a series of experiments to characterize the cell morphology, microfilament rearrangement, focal adhesion formation, nuclear area changes and hypertrophic phenotype of chondrocytes in response to these soft/stiff micropillar substrates. These results provide a deeper explanation of the cell behaviour changes from mechanoreception, mechanotransduction to hypertrophic differentiation in response to the stiffened ECM microenvironment.

## 1 Introduction

Articular cartilage is a highly differentiated connective tissue that links the ends of bones in synovial joints [1]. It serves as an articular head surface with a low friction function and withstands a series of loads, including tensile, compressive and shear forces during motion [2] due to its unique resident chondrocytes and special extracellular matrix (ECM) microenvironmental network structure [3]. Cartilage contains a small proportion of chondrocytes (∼2 ​wt%) but a large proportion of ECM components, including water (∼65–85 ​wt%); collagens (∼20–30 ​wt%), mainly including collagen type II, IX and XI; and aggrecans (∼10–15 ​wt%), which form a well-organized three-dimensional (3D) network that allows cartilage to extraordinarily bear mechanical stimuli [4]. During physiological exercise, cartilage maintains an elegant ECM homeostasis, but this dynamic homeostasis is extremely easily disrupted in the face of any excessive mechanical loads, acute trauma and age-related degeneration [5,6]. For instance, Li et al. reported that the abnormal expression of tenascin-C, a macromolecular glycoprotein, would change the adhesion of ECM and the transduction of mechanical signals, leading to the inactivation of Hippo/YAP signals, thus enhancing endochondral ossification and eventually leading to the occurrence of bone and joint diseases [7]. Moreover, because of the lack of blood vessels, lymphatic vessels and nerves, damaged cartilage has little capacity to repair [8] and has a great possibility of developing into a state of osteoarthritis (OA), a disease that affects all joint tissues and ultimately leads to joint pain, impaired joint function and even disability [9]. Therefore, interpreting the biomechanisms form mechanoperception to chondrocyte behaviour at the cellular level and seeking targeted therapies for cartilage injury at the tissue level have become great challenges.

The mechanical properties of cartilage are determined by the composition and structure of its ECM. Chondrocytes secrete and deposit tissue-specific collagens including types II, III, VI, IX, X, XI, XII and XIV, and form the primary fibre-like 3D framework of the cartilage matrix [10]. The three major collagen types, i.e., II, IX and XI, covalently cross-link with each other to form a series of heteropolymeric fibrils based on the intermolecular bonds of trivalent hydroxylysyl pyridinoline (HP) between amino-(N)/carboxy-(C) telopeptides and helical sites [11]. These linkages then trap high concentrations of proteoglycans, including aggrecan, perlecan and lubricin, and glycosaminoglycans (GAGs), including chondroitin sulfate, keratin sulfate, dermatan sulfate and hyaluronan [12], to form an integrated collagen fibrillar network, which compensates for the swelling pressure generated by the osmotic properties of proteoglycans and GAGs and ultimately enables the cartilage to withstand and redistribute mechanical forces [13]. However, this collagen fibrillar network altered in patients that are elderly and have OA disease [14,15]. In patients that are elderly, the accumulation of aggrecan in cartilage is impaired due to an ageing-related imbalance between ECM synthesis and degradation, and the cross-linking of collagen fibres is enhanced due to the accumulation of advanced glycation end products [14]. Jaabar et al. found that collagen fibers in articular cartilage ECM would increase compensatively at the early stage of OA, and this compensation failed when OA developed to the late stage, and the metabolic imbalance of cartilage chondrocytes led to the structural changes and stiffening of ECM [16]. Wen et al. found that the gradual loss of aggrecan and the enhancement of collagen fiber cross-linking increased fibrosis-like collagen bundles, weakened the ability to resist mechanical stimulation, reduced the elasticity of fibril network, and finally led to OA [17]. In addition, Stolz's AFM nano-indentation test proved that the changes in supramolecular compounds at the level of individual collagen fibrils at the nano-scale are correlated with the nanomechanical properties of cartilage in the progression of OA [18]. Other reports also indicate a total stiffening effect of the cartilage ECM that occurs in the transition from healthy to OA tissues by characterizing an increase in Young's moduli and an increase in Mankin scores [16,19]. However, even after decades of mechanobiological research, the role of OA-associated ECM stiffness in cell behaviour and its underlying biomechanisms remains largely unknown.

Chondrocytes are a unique cell type in all kinds of cartilage. They occupy less than 10% of the volume of the entire cartilage, but are responsible for the maintenance of cartilage ECM composition [20], the integrity of cartilage morphology [21] and the remodelling of the chondrocyte niche [22]. Chondrocytes are considered a typical kind of mechanosensitive cell because they live in a constantly changing mechanical microenvironment throughout their life [23]. They can first change the structure and morphology of the cytoskeleton through mechanoreception and trigger mechanotransduction by changing a series of subcellular units, including stretch-activated ion channels, integrins, focal adhesion plaques and intracellular organelles [24]. In mechanotransduction, a key step is the conversion of physical input into a chemical signalling cascade by chondrocytes, which determines cell behaviour, including cell viability, adhesion, migration, proliferation, differentiation, apoptosis, cell cycle and cell communication [25,26].

In the current study, we designed a novel patterned equidistant micropillar substrate based on polydimethylsiloxane (PDMS) and optimized the minimum size of fibre-like equidistant micropillars to mimic the collagen fibrillar network of the cartilage matrix. After changing the curing component of these micropillars, we fabricated micropillar substrates with the same topology but different stiffnesses to mimic the stiffening of the matrix during the transition from normal to OA. We investigated the cell behaviour changes of chondrocytes in response to these fibre-like micropillar substrates with different stiffnesses and aimed to interpret the real cell fate and its underlying biological mechanism in the ECM stiffening that occurs with osteoarthritis disease.

## 2 Methods and materials

### 2.1 Conventional reagents

Unless otherwise stated, the conventional chemical reagents used in the study were purchased from Sigma-Aldrich (St. Louis, MO). From Abcam (Cambridge, UK), the antibodies include: *Anti*-ColX (ab182563); *anti*-Osx/Sp7 (ab209484); Erk1/2 (ab17942); *p*-Erk1/2 (Erk1 (pT202) ​+ ​Erk2 (pT185/), ab76299); Vinculin (ab129002). The secondary antibodies include: Goat Anti-Mouse IgG H&L (Alexa Fluor® 488) (ab150113), Goat Anti-Mouse IgG H&L (Alexa Fluor® 647) (ab150115), Donkey Anti-Rabbit IgG H&L (Alexa Fluor® 488) (ab150073), Donkey Anti-Rabbit IgG H&L (Alexa Fluor® 647) (ab150075). From Santa Cruz (Delaware Avenue, CA): the primary antibody is *anti*-β-actin (sc-47778) and the secondary antibodies include anti-mouse (*m*-IgGκ BP-HRP, sc-516,102) and anti-rabbit (sc-2357). From ZEN BIO (Chengdu, China): anti-Col1a2 (343,277); *anti*-MMP13 (820,098); *anti*-JNK (R22866); *anti*-pJNK (Thr183, 381,100); *anti*-p38 (200,782); *anti*-p-p38 (Thr180/Tyr182, 310,091). FITC-labeled phalloidin (Alexa Fluor® 488 phalloidin, A12379, Invitrogen) was purchased from Invitrogen. The other protein regents include: the protein sample buffer (Laemmli Sample Buffer, 2 ​× ​, 1,610,737, Bio-Rad, Hercules, CA); The blotting reagent (luminol reagent, sc-2048, Santa Cruz). Polyvinylidene difluoride (PVDF, Immobilon®, Sandwiches, Billerica, MA). The gene reagents included: the mRNA extract kit (74,136, Qiagen, Valencia, CA); DNAse I enzyme (Glen Burnie, Mbi, MD); cDNA synthesis kit (RevertAid H Minus First Strand cDNA Synthesis Kit, K1632, Thermo Fisher); and qPCR kit (SYBR® Premix Ex TaqTM II, RR82WR, TaKaRa, Tokyo, Japan).

### 2.2 Design and fabrication of patterned equidistant micropillar substrates

**Fig. 1:**
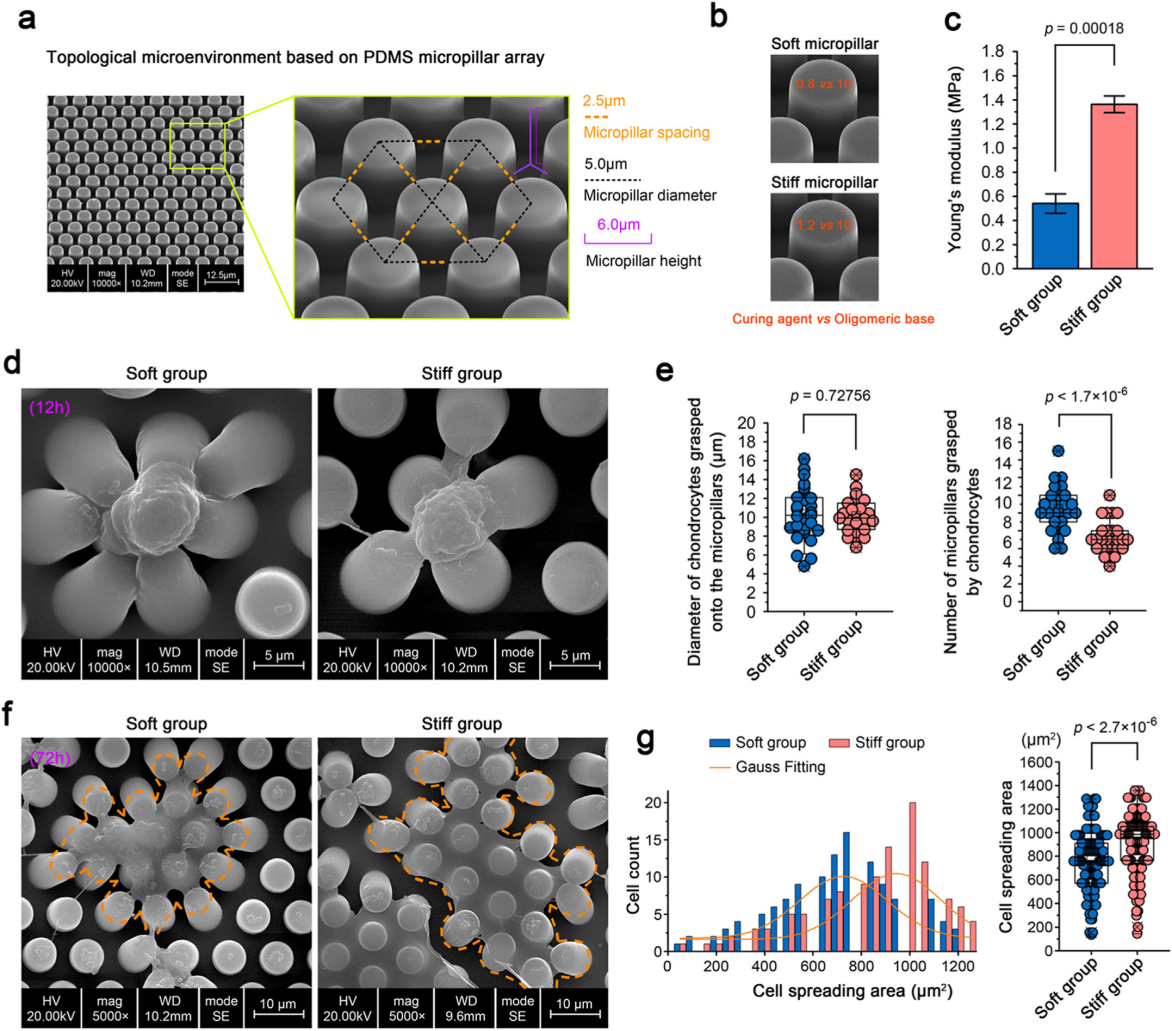
Cell morphological changes of chondrocytes in response to PDMS-patterned equidistant micropillar substrates with different stiffnesses. **a).** SEM image indicating the cell substrate with patterned equidistant micropillars based on PDMS. Any two micropillars were evenly spaced. The specific parameters of the micropillars are labeled on the right. **b).** SEM images showing patterned equidistant micropillars with different stiffnesses attained by changing the ratio of curing agent to oligomeric base. **c).** Histogram showing the change in Young's moduli between the soft and stiff micropillars. The data were based on at least 3 independent experiments (n ​≥ ​3). **d).** Representative SEM images indicating the morphological changes when chondrocytes were seeded onto patterned equidistant micropillar substrates with different stiffnesses for 12 ​h. The images were chosen based on 5 independent experiments (n ​= ​5). **e).** Statistical data showing that the cell size of chondrocytes did not change (left), but the number of individual chondrocytes grasping the micropillars changed (right), within a short time of cell seeding (12 ​h). The statistical data were based on 27 ​cells from 5 independent experiments. **f).** Representative SEM images indicating the morphological changes when chondrocytes were seeded onto patterned equidistant micropillar substrates with different stiffnesses for 72 ​h. The images were chosen based on 10 independent experiments (n ​= ​10). **g).** Cell spreading area changes of chondrocytes seeded onto patterned equidistant micropillar substrates with different stiffnesses for 72 ​h. We counted 118 ​cells from 10 independent experiments to show the changes of cell spreading areas. The data presented in **e** and **g** are shown as box (from 25, 50–75%) and whisker (SD values) plots. The significant data presented in **c**, **e** and **g** are based on Two-tailed Student's t Tests (source data).

In order to mimick the collagen fibrillar network of cartilage matrix, we designed a novel kind of fiber-like equidistant micropillar substrates based on polydimethylsiloxane (PDMS) as described in Fig. 1a. The radius of micropillar was set at 2.5 ​μm; the spacing between any two micropillars was 2.5 ​μm; and the height of the micropillars was 6 ​μm. These micropillars are obtained by peeling off the disposable SU-8 structured wafer after molding. We can obtain micropillars with radius of 1 ​μm, but its stability is not as good as that of 2.5 ​μm. Thus, we applied the micropillars with a radius of 2.5 ​μm in the study. To obtain patterned equidistant micropillar substrates with different stiffnesses, we changes the curing ratio (crosslinker of Sylgard184) to PDMS base: 0.8:10 and 1.2:10) to form soft and stiff micropillar substrates. After sterilization at UV radiation for 1 ​h and coating with dopamine solution (0.12 ​mg/ml in 1 ​mg/ml Tris) for 30 ​min, the soft and stiff micropillar substrates were prepared for chondrocyte seeding.

### 2.3 Measurement of Young's modulus of patterned equidistant micropillar substrates

The measurement of Young's moduli were based on ElectroForce 3100 test instrument (Bose, Shanghai, China). The specific parameters were shown previously [27,28]: The tensile strength of the materials of micropillar substrates was measured by an Instron universal testing instrument (Instron 5567, Instron, USA) at a stretching rate of 200 ​mm/min. The Young's modulus was obtained from linear slopes of the tensile stress-strain curves within 10% strain. All results for each sample were the average of five specimens.

### 2.4 Chondrocyte isolation and culture

Firstly, the cartilage tissues were procured in accordance with ethical principles, and the usage protocol was finally approved by IRB at the West China Hospital of Stomatology (WCHSIRB-OT-2020-048) prior to the initiation of the experiments. Chondrocyte isolation and culture were originated from the primary isolation methods [29,30]. The cartilage tissues were washed with 1 ​× ​sterile PBS (containing 5% antibiotics), and trypsinized in protease solution (0.25%, v/v) for 20–30 ​min at room temperature (RT) and then digested in type II collagenase (0.5%, w/v, Sigma-Aldrich) for overnight. The isolated chondrocytes were collected in fresh 10% FBS DMEM after removal of protease and collagenase solution. The re-suspended cells were then seeded onto petri dishes and incubated with 5% CO_2_ at 37 ​°C. Passages 1 were mainly used in the experiments.

### 2.5 Cell seeding

After the preparation of patterned equidistant micropillar substrates, chondrocytes were seeded onto them in 10% FBS DMEM. For detection of qPCR, RNA sequencing, western blotting and immunofluorescence, chondrocytes need to be seeded and equilibrated for 12 ​h and then be starved in 2% FBS DMEM media for 12 ​h. Then the media were changed to 1% FBS DMEM and collected cell samples according to the requirements of the specific experiments. For SEM test, we directly collected cell samples at 12 and 72 ​h after seeding. In terms of the number of chondrocytes seeded onto the micropillar substrates, the cell density was different in different experiments. For gene detection (qPCR and RNA sequencing) and protein detection (western blotting), each substrate (2.5 ​× ​2.5 ​cm^2^) was seeded with 5.5 ​× ​10^5^ ​cells; For cell immunofluorescence, each substrate (0.5 ​× ​0.5 ​cm^2^) was seeded with 2000 ​cells.

### 2.6 Scanning electron microscope (SEM) test

We have already demonstrated the SEM test for both seeded cells [31] and tissues [32] previously. In brief, for patterned equidistant micropillar substrates with different stiffnesses, we directly scanned the substrates after spouting a layer of gold powder onto them. For chondrocytes seeded onto these substrates, we firstly fixed the cells with glutaraldehyde solution (2.5%, v/v) for 1–2 ​h, and then dehydrated the cells in a gradient from 50% to 100% ethanol. We next coated the dehydrated chondrocytes with a thin layer of gold powder, and finally scanned by SEM.

### 2.7 RNA sequencing

Chondrocytes were allowed to seed onto patterned equidistant micropillar substrates with different stiffnesses (total 5.5 ​× ​10^5^ ​cells at 2.5 ​× ​2.5 ​cm substrate areas). The cells were allowed 12 ​h to attach, and starved in low-FBS (2%) DMEM for another 12 ​h. Next, chondrocytes were maintained in 1% FBS DMEM for 48 ​h, and the cell lysates were obtained by using Trizol (15,596–026, Thermo Fisher). RNA sequencing was achieved by Shanghai Lifegenes (Shanghai, China) after assessment for RNA integrity (Nano 6000 Kit for Bioanalyzer 2100 system, Agilent Technologies, CA). The index-coded lysates were clustered (HiSeq 4000 ​PE Cluster kit, Illumina, San Diego, CA) by using a cBot Cluster Generation System. The raw data in fast q format were obtained via in-house Perl scripts. By summing the FPKMs of transcripts in every genome, gene FPKMs were figured. To test the statistical differences of gene expressions in their pathways, we applied the software KOBAS v3.0. KEGG terms with |FoldChange| ≥1.5 and p value ​< ​0.05 to distinguish the different expressed genes.

### 2.8 Quantitative real-time PCR (qPCR)

Chondrocytes were allowed to seed onto patterned equidistant micropillar substrates with different stiffnesses (total 5.5 ​× ​10^5^ ​cells at 2.5 ​× ​2.5 ​cm substrate areas). Then cells were equilibrated for 12 ​h, and then starved for 12 ​h by low-FBS (2%) DMEM culture media. After culturing for 48 ​h in fresh 1% FBS DMEM, RNAs were collected by employing the RNA Isolation Kit (RP5611, Biotech, Peking, China). Then RNAs were treated with DNAzyme I (Mbi, Glen Burnie, MD) to remove any interference of DNA fragments. For cDNA synthesis, 5 ​μg of total RNA from each sample was used to synthesize first-strand cDNA forming a volume of 20 ​μl cDNA solution according to the manufacturer's protocol (K1632, Thermo Fisher).

To detect gene changes, qPCR was utilized with a 25 ​μl system (the mixture solution with SYBR Green I: 12.5 ​μl; forward primer: 1 ​μl; reverse primer 1 ​μl; cDNA template: 1 ​μl; and DDH_2_O: 9.5 ​μl). The reaction steps included a pre-denaturation (95 ​°C, 5 ​min), and a 45 cycles of melting (95 ​°C, 5 ​s), tempering (60 ​°C, 5 ​s), and extension (72 ​°C, 5 ​s). The fold changes were calculated based on cycle threshold (CT). Glyceraldehydes-3-phosphate dehydrogenase (GAPDH) worked as the inner control. The primer pairs were designed in Table 1.

**Table 1:** Primer pairs designed in the study.

Protein name	Gene name/gene ID	Primer pairs
Glyceraldehyde-3-phosphate dehydrogenase	GAPDH (136bp) (NM_001289726.2)	Forward: TGAGCCTCCTCCAATTCAACC Reverse: AAATCCGTTCACACCGACCT
Collagen type I, alpha 2	Col1a2 (154bp) (NM_007743.3)	Forward: CCCATGAACATTCGCACCAC Forward: CCTCAGTTCGTGTCAGCCTT
Collagen type X, alpha 1	Col10a1 (193bp)	Forward: TGCTAACCACGGGGTAACAG
	(NM_009925.4)	Reverse: CACATGCACGTGGTAGGAGA
Osterix/transcription factor 7	Osx/SP7 (127bp)	Forward: GATGGCGTCCTCTCTGCTTG
	(NM_130458.4)	Reverse: TCCTTTCCCCAGGGTTGTTG
Matrix metallopeptidase 13	MMP13 (119bp)	Forward: CAGTTGACAGGCTCCGAGAA
	(NM_008607.2)	Reverse: TTCACCCACATCAGGCACTC

### 2.9 Western blotting

Western blotting protocol was followed by previous usage [33]. Chondrocytes were seeded onto patterned equidistant micropillar substrates with different stiffnesses (total 5.5 ​× ​10^5^ ​cells at 2.5 ​× ​2.5 ​cm substrate areas). The cells were allowed 12 ​h to attach, and 12 ​h to starve in 2% FBS-DMEM. Then, cells were maintained in fresh 1% FBS DMEM for 48 ​h. The cell samples were collected in 1.5 ​ml tubes (sterile Rnase-free, Coring), total lysate protein was obtained by RIPA (P0013C, Beyotime, Guangzhou, China) containing PMSF (P7626, Sigma) with 1% content. Quantification was performed with a BCA kit (Beyotime) and boiled in protein sample buffer (1,610,737, Bio-Rad) at a 1:1 ratio for 5 ​min. The proteins were separated by using 10% SDS-PAGE for 2 ​h and transferred to PVDF membrane for 2 ​h. After blockage with skim milk (5%, w/v) for 1 ​h at RT, PVDF membrane was incubated with the primary antibodies (1:1000 for Abcam antibody, 1:500 for Santa Cruz, and 1:2500 for other companies, depending on specific antibody properties) overnight. The secondary antibodies (1:1000 anti-mouse and 1:3000 anti-rabbit, Santa Cruz) were used the next day, depending on the source of the primary antibodies. Immunoreactive blotting images were logged by adopting blotting reagent (sc-2048, Santa Cruz).

### 2.10 Immunofluorescence based on confocal laser scanning microscopy (CLSM)

Chondrocytes were allowed to seed onto patterned equidistant micropillar substrates with different stiffnesses (total 2000 ​cells at 0.5 ​× ​0.5 ​cm substrate areas). To directly observe chondrocyte morphological changes, we did not starve cells. After seeding for 72 ​h, chondrocyte samples were collected for immunofluorescence. The immunofluorescent protocol was previously described [34]. In short, chondrocytes were fixed with paraformaldehyde (PFA, 4%, w/v) for 5–10 ​min, and then permeated with Triton X-100 (0.5%, v/v) for 5 ​min. The cells were required to wash three times in 1 ​× ​PBS before milk blockage in 5% BSA for 2 ​h. Then, the cells were washed and incubated with the primary antibody (1:200 dilution) at 4 ​°C overnight. Then the cells were added with the secondary antibody (AlexaFluor647, 1:400 dilution) for 2 ​h at RT. Finally, the cells were counterstained with F-actin (phalloidin, FITC-labeled) and Dapi (sigma). The immunofluorescent images were obtained through CLSM (Olympus, FV3000, Japan).

### 2.11 Osteoarthritis (OA) mouse model

Osteoarthritis (OA) mouse model was established by anterior cruciate ligament transection (ACLT) surgery based on 8-week-old C57 mice as previously described [3]. ACLT surgery was performed on the right knee joints and the left knee joints used as sham controls. All ACLT procedures were reviewed and approved by IRB and were strictly followed by stand surgery [35]. After 4 and 12 week's post surgery, the mice were sacrificed and the knee joint samples were collected entirely. The samples at 4 week's post surgery were used for SEM detection and the ones at 12 week's post surgery were used for histological and immunohistochemical analysis.

### 2.12 HE staining & immunohistochemistry

HE staining & immunohistochemistry was done by using paraffin section of knee joint cartilage tissues as previously described [3,9]. Briefly and importantly, for HE staining, during the hematoxylin and the eosin staining, color separation should be achieved by treatment of HCL-EOTH and ammonia-H_2_O. For immunohistochemistry, the primary antibodies including Col10a1, Col1a2, Osx and MMP13 were used at 1:200 dilution, and the secondary antibodies were used at 1:200 dilution. The immunohistochemical staining was achieved by using a kit (VECTASTAIN®Elite®ABC Kit for Mouse IgG, PK-6102, & Rabbit IgG, PK-6101, Vector Laboratories, Burlingame). The images were obtained by scanning system (Olympus, VS200, Japan).

### 2.13 Statistical analysis

Data presented in this study were as mean ​± ​SD with at least biological triplicates (n ​≥ ​3). The statistical analyses were relied on Two-tailed Student T-test for differences between any two groups and one/two-way analysis of variance (ANOVA) for differences of multi-groups. All statistical data analyses were attached in source data file. Post-hoc analysis employed Fisher's protected least significant differences (PLSD) and the threshold of significant levels were set at 0.05 in each analysis.

## 3 Results

### 3.1 Cell morphological changes of chondrocytes in response to patterned equidistant micropillars with different stiffnesses

Based on the typical meshwork structure of collagen fibres in the cartilage matrix [10,36], we designed novel PDMS-patterned equidistant micropillars to mimic this microenvironmental topology based on our previous experiments [37,38] (Fig. 1a). The height of these micropillars was 6 ​μm, the radius of these micropillars was 2.5 ​μm, and the distance between any two neighbouring micropillars was 2.5 ​μm, ensuring the equidistant meshwork-like property. We then changed the curing ratio of the micropillars (curing agent (Sylgard184) to oligomeric base: 0.8:10 and 1.2:10 in this study), and thus fabricated micropillars with soft and stiff stiffnesses, respectively (Fig. 1b). These soft and stiff micropillars had the same topology but different Young's moduli (Fig. 1b & c). In this way, we obtained two kinds of cell substrates, which had the same patterned equidistant micropillar structures that mimicked the meshwork structure of collagen fibres in the cartilage matrix but different stiffnesses. We seeded chondrocytes onto these patterned equidistant micropillar substrates with soft/stiff stiffnesses to investigate the cell morphological changes (Fig. 1d–g). At the initial 12 ​h after seeding, we did not observe changes in cell size, but interestingly, we found that the round chondrocytes were able to adhere and grasp more micropillars on the soft substrate than on the stiff substrate (Fig. 1d). Statistical analyses of cell size and chondrocyte-grasped micropillar numbers (Fig. 1e) also confirmed these results. At 72 ​h after seeding, we observed that the chondrocytes were spreading out in flake-like manner. However, the spreading area of chondrocytes on the stiff substrate was much larger than that on the soft substrate. Moreover, the capacity of chondrocytes to bend these covered micropillars was also completely different. On the soft substrate, chondrocytes could bend more micropillars (Fig. 1f). Quantitative analysis demonstrated the difference in chondrocytes in terms of the spreading area (Fig. 1g). Taken together, these results indicated the cell morphological changes of chondrocytes in response to patterned equidistant micropillar substrates with soft/stiff stiffnesses.

### 3.2 Changes in the cytoskeleton-focal adhesion axis of chondrocytes in response to patterned equidistant micropillars with different stiffnesses

**Fig. 2:**
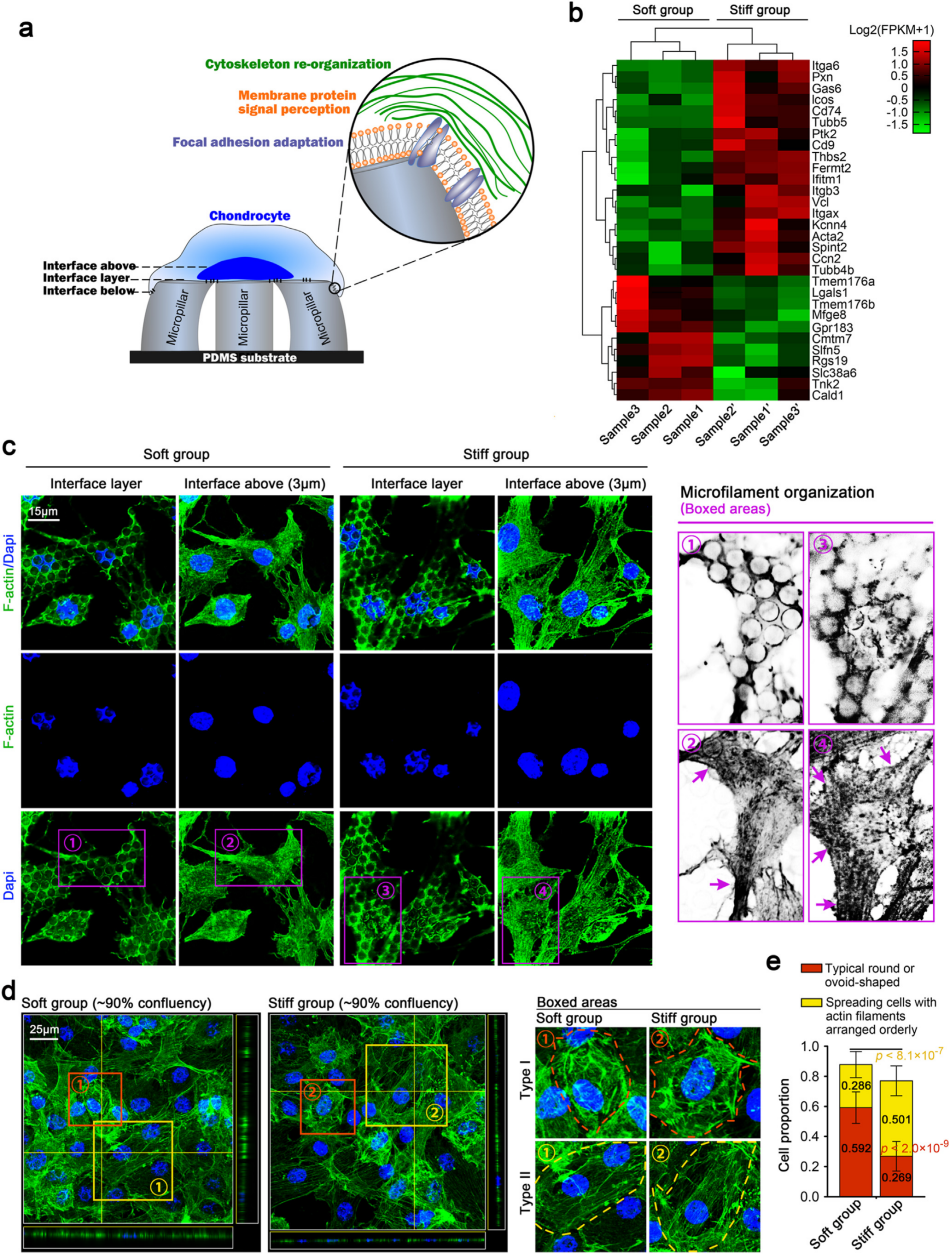
Cytoskeleton changes of chondrocytes in response to patterned equidistant micropillar substrates with different stiffnesses. **a).** Schematic diagram indicating the mechanosensitive changes of chondrocytes in response to patterned equidistant micropillars with different stiffnesses. These mechanosensitive changes included but were not limited to cytoskeleton reorganization, membrane protein signal perception and focal adhesion adaptation. **b).** Pheatmap showing the top 30 gene changes in cytoskeleton mediation, membrane signal perception and focal adhesion adaptation of chondrocytes in response to patterned equidistant micropillar substrates with different stiffnesses. Samples 1 and 1′, 2 and 2′, and 3 and 3′, were from the same mother cells. The changes were based on the original FPKM data. FPKM, fragments per kilobase of exon model per million mapped fragments. **c).** Representative CLSM images showing cytoskeleton changes of individual chondrocytes in response to patterned equidistant micropillar substrates with different stiffnesses by characterizing microfilament (F-actin) organization. “Interface layer” indicates the contact interface between chondrocytes and top surface of micropillars; “Interface above (3 ​μm)” indicates 3 ​μm above the interface (cellular layer); and boxed areas in purple indicate the distribution of microfilaments. The images were based on three independent experiments (n ​= ​3). **d).** Representative CLSM images indicating cytoskeleton changes of full confluence chondrocytes in response to patterned equidistant micropillar substrates with different stiffnesses by characterizing microfilament (F-actin) organization. Boxed areas in red indicate the typical circular or ovoid-shaped cells, and boxed areas in yellow indicate the spreading cells with actin filaments arranged in an orderly manner. The images were based on three independent experiments (n ​= ​3). **e).** Histogram showing the different cell proportions of the typical circular or ovoid-shaped cells and the spreading cells with actin filaments arranged in an orderly manner in response to patterned equidistant micropillar substrate with different stiffnesses. The significance data are based on Two-tailed Student's t Tests (source data).

To further investigate the mechanoperception of chondrocytes in response to the patterned equidistant micropillar substrates with soft/stiff substrates, we focused on the changes in the cellular membrane, cytoskeleton and focal adhesion plaque that occurred during the interactions between chondrocytes and these micropillars with tuneable stiffness (Fig. 2a). We sorted the top 30 gene candidates of the cellular membrane, cytoskeleton and focal adhesion plaque from RNA sequencing and clustered them in a pheatmap (Fig. 2b). From this result, we found an increase in the number of genes in chondrocytes in response to the stiff patterned equidistant micropillar substrates.

We detected cytoskeletal changes by characterizing the distribution of microfilament organization. In individual chondrocytes, we showed cytoskeletal changes in the following two layers. One was the interface layer where the cell just contacted the top surface of the micropillars (Fig. 2c, interface layer, equivalent to the adhesive bottom layer of chondrocytes). The cells at this layer displayed a uniform meshwork-like distribution of cytoskeleton after F-actin staining, and the cytoskeleton on the stiff substrate covered a greater meshwork area, indicating that the cell spreading area was larger than that on the soft substrate. Moreover, the nuclei also showed a meshwork-like distribution at this layer (the specific difference in nuclei is detailed in Fig. 5). The other layer was the interface above (∼3 ​μm from the contact surface layer, almost equivalent to the top view of chondrocytes on conventional culture dishes), and we found that the cytoskeleton on the stiff substrate displayed an increasingly more orderly microfilament arrangement than that on the soft substrate (Fig. 2c, left). In particular, from the boxed areas in purple, the enhanced arrangement of the actin microfilaments of chondrocytes on the stiff substrate was confirmed (Fig. 2c, right). In chondrocytes with a confluency greater than 90%, we detected an interesting result regarding the cell morphology based on a collective of chondrocytes by layer-scanned CLSM (Fig. 2d & e). We found that the largest difference was in the proportion of cell morphologies (round or ovoid-shaped morphology (boxed in red) *vs.* the spreading morphology with aligned actin filaments (boxed in yellow), Fig. 2d). We used CLSM layer scanning and 3D image stacking technology and established a XYZ three-dimensional space image, which allowed us to observe the morphological changes of chondrocytes on PDMS micropillar more intuitively and stereoscopically. XZ view refers to the horizontal section of chondrocytes in a field of view taken by CLSM. YZ view refers to the vertical section of chondrocytes in a field of view taken by CLSM. Through this 3D image stacking, the upper interface could be observed clearly. Additionally, in both directions we could observe not only the spreading of chondrocytes on the PDMS micropillar, but also the morphological changes of the corresponding chondrocyte nuclei on different micropillar substrates (as shown in Fig. 5). Here we found that more round or ovoid-shaped chondrocytes formed on the soft micropillar substrate, and more spreading cells with enhanced aligned actin filaments accumulated on the stiff micropillar substrate. By statistical analysis, we confirmed that on the soft substrate, round or ovoid-shaped chondrocytes accounted for 59.2% of the chondrocytes, and spreading cells with aligned actin filaments accounted for 28.6%, while on the stiff substrate, round or ovoid-shaped chondrocytes only accounted for 26.9%, and spreading cells with aligned actin filaments accounted for 50.1% (Fig. 2e).

**Fig. 3:**
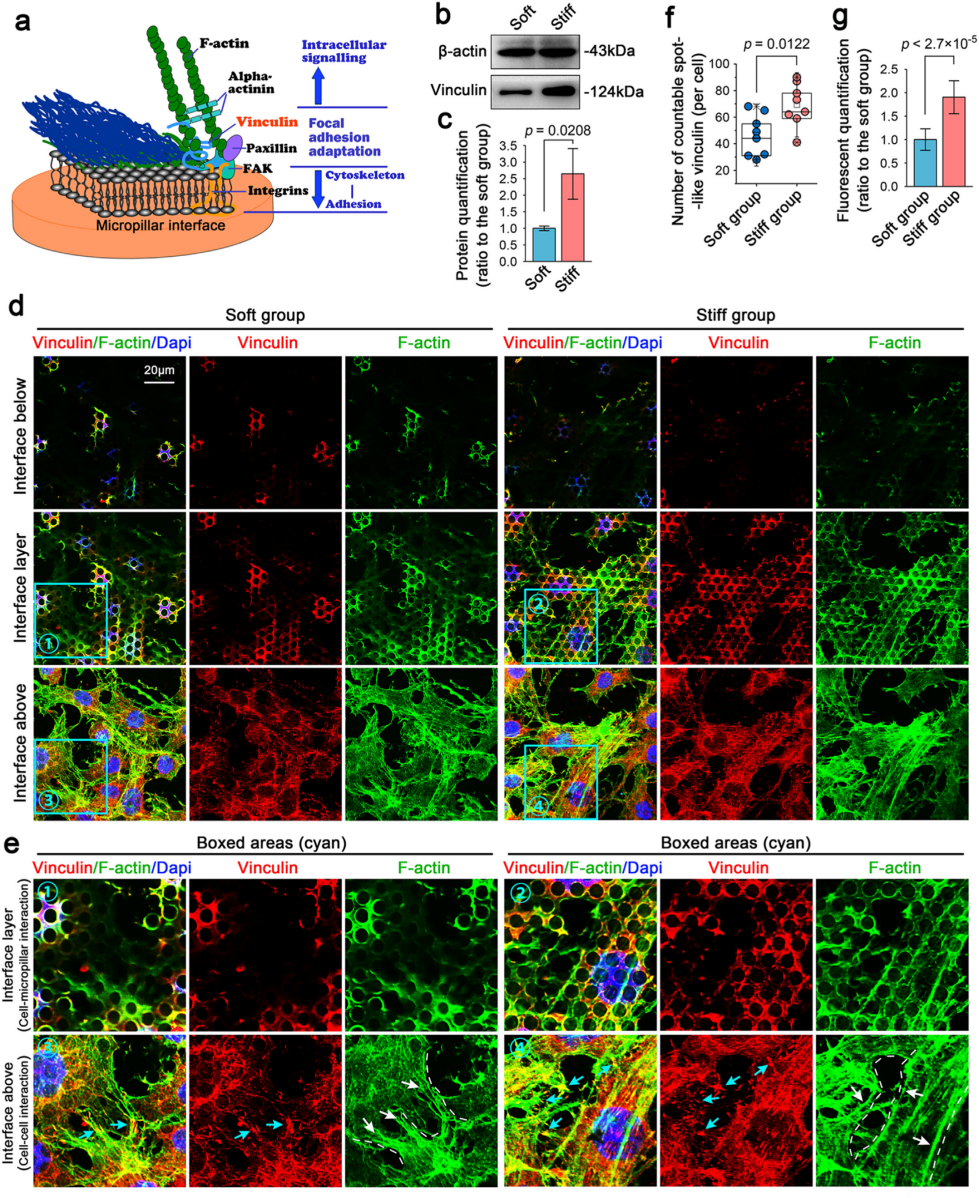
Changes of vinculin in chondrocytes in response to patterned equidistant micropillar substrates with different stiffnesses. **a).** Schematic diagram indicating the mechanosensing and mechanotransduction at the membrane-cytoskeleton-cell adhesion axis in chondrocytes in response to patterned equidistant micropillars with different stiffnesses. **b).** Western blotting indicating the protein changes of vinculin in chondrocytes in response to patterned equidistant micropillar substrates with different stiffnesses. The images were based on three independent experiments (n ​= ​3). **c).** Quantification of vinculin in (b). The data were based on three different independent experiments (n ​= ​3). **d).** Representative CLSM images indicating the distribution changes of vinculin in chondrocytes in response to patterned equidistant micropillar substrates with different stiffnesses. “Interface below”, indicates fluorescence that was captured by CLSM under the cells deep into the micropillars; “Interface layer” indicates fluorescence that focused on the contacted interface; and “Interface above” indicates fluorescence that reflected the whole cell morphology of chondrocytes grasped micropillars. The images were based on three independent experiments (n ​= ​3). **e).** Boxed areas further indicating the distribution of vinculin in chondrocytes in response to patterned equidistant micropillar substrates with different stiffnesses. Cell-micropillar interactions were directly shown at the interface layer; and cell-cell interactions were observed above the interface layer. The arrows in cyan indicate the vinculin-formed plaques, and the arrows in white indicate the microfilament distribution. **f).** Quantification of the number of spot-like distribution of vinculin per chondrocytes in response to patterned equidistant micropillar substrates with different stiffnesses. The analysis was based on eight cells from three independent experiments (n ​= ​8). **g).** Quantification of total fluorescent optical density in vinculin per chondrocytes in response to patterned equidistant micropillar substrates with different stiffnesses. The analysis was based on eight cells from three independent experiments (n ​= ​8).

Focal adhesion plaques are involved in the linkage of integrin adhesion proteins to the actin-cytoskeleton [39] and in the intracellular signal conversion and transduction [40] (Fig. 3a). Based on the RNA sequencing results shown in Fig. 2b, we detected changes of vinculin, an important adhesion protein in the membrane-cytoskeletal system, of chondrocytes in response to patterned equidistant micropillar substrates with soft/stiff micropillar substrates. We detected its total protein expression by western blotting and found that it was expressed in chondrocytes at higher level on the stiff substrate than on the soft substrate (Fig. 3b). Quantitative analysis further confirmed this result (Fig. 3c). We next detected its distribution by immunofluorescence (Fig. 3d). At the interface below (upper), when the fluorescence of vinculin was just captured at the bottom layer on the stiff substrate (Fig. 3d, right), we observed partial individual clear cellular fluorescence on the soft substrate (Fig. 3d, left), indicating that chondrocytes pressed down the soft micropillars. At the interface layer (medium), we observed that vinculin was distributed in a meshwork shape, and the meshwork structure on the stiff substrate was larger than that on the soft substrate. At the interface above (lower), we obtained the top view of vinculin distribution in chondrocytes in response to the soft/stiff micropillar substrates and found that the expression of vinculin was much higher in chondrocytes in response to the stiff substrate than in response to the soft substrate. We extracted information from the boxed areas (Fig. 3d, cyan) and showed more detailed expression and distribution changes of vinculin at the interface layer and the interface above (Fig. 3e). At the interface layer, we clearly observed the different meshwork expressions of vinculin in chondrocytes on the micropillar substrates with different stiffnesses, which reflected the direct interaction between cells and the top surface of micropillars (upper). At the interface above, we observed the formed spot-like distribution of vinculin in chondrocytes triggered by both substrates, but the spot number on the stiff substrate was clearly higher than that on the soft substrate, and quantification about the number of spot-like distribution further confirmed the result (Fig. 3f). Moreover, we detected the total fluorescent optical density per cell and quantified the expressions of vinculin in chondrocytes in response to soft/stiff micropillar substrates and this result also indicated high expressions of vinculin in chondrocytes on the stiff substrate (Fig. 3g). In addition, a large portion of the spot-like distributions (cyan arrows) were located along the direction of the cytoskeleton connection between the two cells (white dotted line and white arrows), which indicated that cell-to-cell interaction simultaneously occurred in the presence of cell-matrix interactions between chondrocytes and the top surface of micropillar substrates. Thus, we also detected the expression changes of a vital calmodulin, *E*-cadherin, in chondrocytes in response to the micropillar substrates with different stiffnesses to show the occurrence of cell-to-cell interaction. The result showed that the total protein of *E*-cadherin was higher in chondrocytes on the stiff group than on the soft group (Figs. S1a and b). Moreover, the higher distribution of *E*-cadherin between chondrocytes on the stiff group inferred the enhanced cell-to-cell interaction of chondrocytes relative to that on the soft group (Figs. S1c and d).

Overall, through the changes in the cytoskeleton observed by characterizing actin microfilaments (F-actin) and in the focal adhesion by representing vinculin expression, we indicated the changes in the cytoskeleton-focal adhesion axis in chondrocytes in response to patterned equidistant micropillar substrates with different stiffnesses.

### 3.3 Patterned equidistant micropillars with different stiffnesses trigger Erk1/2/MAPK signalling

**Fig. 4:**
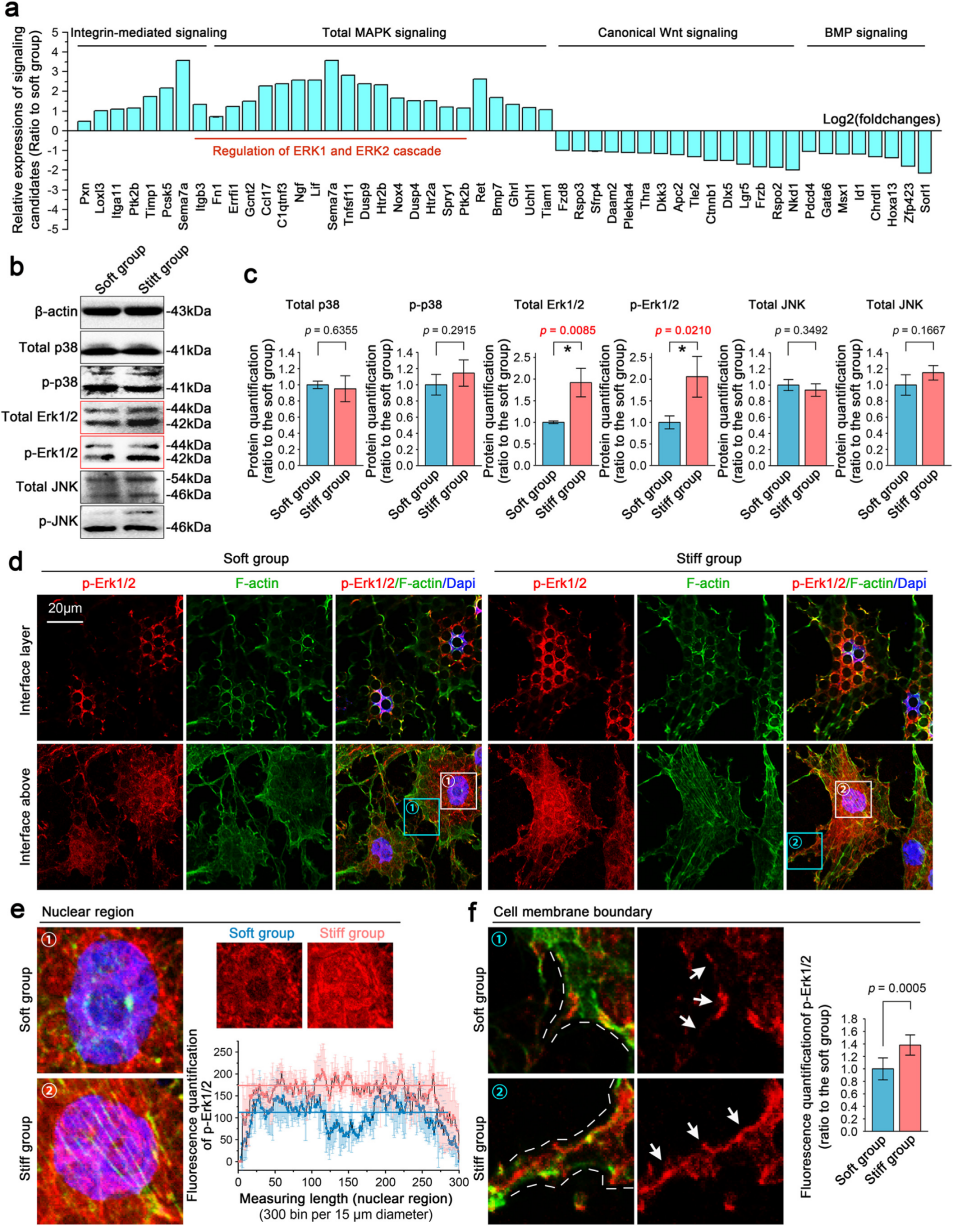
Patterned equidistant micropillar substrates with different stiffnesses activate Erk/MAPK signalling in chondrocytes **a).** Fold changes in all signalling-related genes in chondrocytes in response to the patterned equidistant micropillar substrates with different stiffnesses. The data originated from RNA sequencing. The gene candidates were classified by GO analysis. The fold changes are presented as log2 (foldchange), and the threshold of the p value was set to 0.05. **b).** Western blotting indicating the changes in ERK/MAPK signalling. The images were based on three independent experiments (n ​= ​3). **c).** Quantification of Erk/MAPK proteins in (b). The data were based on three independent experiments (n ​= ​3). **d).** Representative CLSM images indicating the distribution changes of *p*-Erk signalling in chondrocytes in response to patterned equidistant micropillar substrates with different stiffnesses. The images were chosen based on three independent experiments (n ​= ​3). **e).** Nuclear distribution changes in *p*-Erk signalling in chondrocytes in response to patterned equidistant micropillar substrates with different stiffnesses. The nuclear regions originated from the boxed areas in (d, white). Linear fluorescence quantitative analysis was performed to confirm the changes in the nuclear region. **f).** The distribution of Erk/MAPK signalling at the cell membrane boundary in chondrocytes in response to patterned equidistant micropillar substrates with different stiffnesses. The cell membrane boundary regions originated from the boxed areas in (d, cyan). Fluorescence quantitative analysis was performed to confirm the changes in Erk/MAPK signalling at the cell membrane boundary (right). The significant data presented in **c** and **f** are based on Two-tailed Student's t Tests (source data).

We sorted out the changes in four main signalling pathways, i.e., integrin, MAPK, canonical Wnt and BMP signalling pathways, based on Gene Ontology (GO) analysis (attached source data in Fig. 4a). We clustered all changed genes related to these signalling pathways and plotted them in a histogram (Fig. 4a). The results indicated that the changed gene number involved in MAPK signalling was the largest; moreover, the changed gene number of Erk occupied most of the MAPK signalling pathway. We then determined the expression of MAPK signalling components, including p-38, Erk and JNK, by western blotting (Fig. 4b) and found that, regardless of the total amount or phosphorylation, the changes in Erk1/2 were significantly different (Fig. 4c). We next detected the distribution of *p*-Erk1/2 in chondrocytes in response to micropillar substrates with different stiffnesses (Fig. 4d–f). At both the interface layer (upper) and the interface above (lower), we observed that *p*-Erk1/2 was widely distributed in cells, especially in and around the nuclei and at the boundary of the cell membrane. The distribution of *p*-Erk1/2 in these areas was higher in chondrocytes on the stiff micropillar substrate than in those on the soft micropillar substrate (Fig. 4d). Furthermore, we extracted information on *p*-Erk1/2 from the boxed areas (Fig. 4d, white) and analyzed its differential expression in the nuclear region (Fig. 4e). The results showed that the expression of *p*-Erk1/2 on the stiff substrate was higher than that on the soft substrate (Immunofluorescence image, left, and linear fluorescence quantification, lower right). We also analyzed the differential expression of *p*-Erk1/2 ​at the boundary of the cell membrane (Fig. 4e-cyan boxes & 4f) and found that the distribution of *p*-Erk1/2 on the stiff substrate was much higher than that on the soft substrate (Fig. 4f, indicated by white arrows, middle, and quantitative analysis, right).

**Fig. 5:**
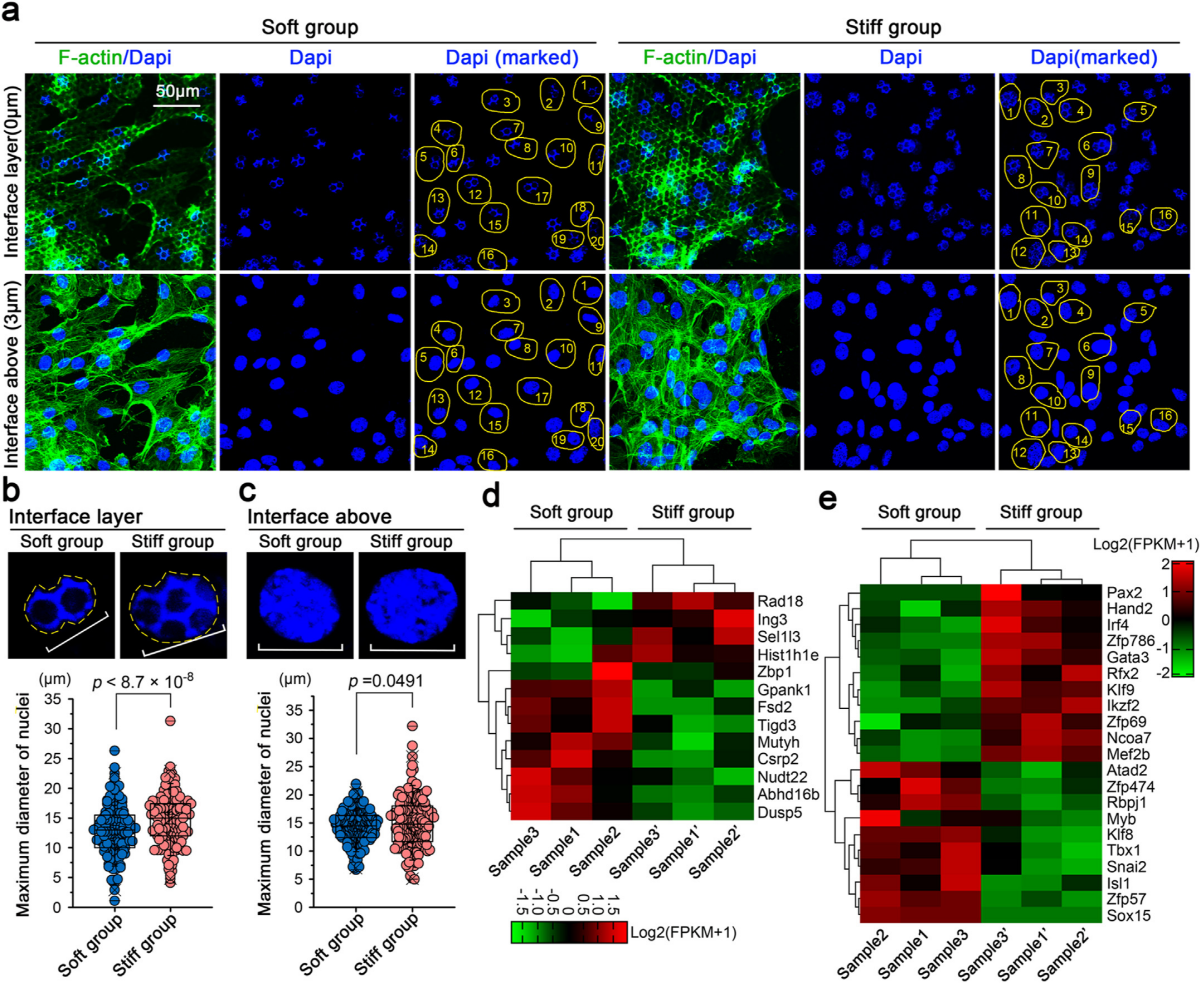
Changes in nuclear spreading areas at the interface layer in chondrocytes in response to patterned equidistant micropillar substrates with different stiffnesses. **a).** Representative CLSM images indicating the enlarged nuclear spreading areas in the stiff group. The images were chosen based on five independent experiments (n ​= ​5). The yellow circle at the interface layer indicates the changes in nuclear spreading areas. **b).** Statistical analysis showing the changes in nuclear spreading areas at the interface layer in chondrocytes in response to patterned equidistant micropillar substrates with different stiffnesses. The data were based on 200 individual nuclei from five independent experiments (n ​= ​5). **c).** Statistical analysis showing the changes in nuclear spreading areas at the interface above layer in chondrocytes in response to patterned equidistant micropillar substrates with different stiffnesses. The data were based on 200 individual nuclei from five independent experiments (n ​= ​5). **d).** Pheatmap showing the changes in gene candidates directly related to nuclear mediation. The data originated from RNA sequencing. Samples 1 and 1′, 2 and 2′, and 3 and 3′, were from the same mother cells. The data were presented based on FPKM (fragments per kilobase of exon model per million mapped fragments). **e).** Pheatmap showing the changes of transcription factors in chondrocytes in response to the patterned equidistant micropillar substrates with different stiffnesses. The data originated from RNA sequencing. Samples 1 and 1′, 2 and 2′, and 3 and 3′, were from the same mother cells. The data were presented based on FPKM. The data presented in **b** and **c** are shown as box (from 25, 50–75%) and whisker (SD values) plots. The significant data presented in **b** and **c** are based on two-tailed Student's t tests (source data).

### 3.4 Patterned equidistant stiff micropillars enlarge the nuclear spreading area of chondrocytes at the interface layer

Cytoplasmic signalling occurs in the nucleus to regulate gene transcription and ultimately achieve cell behaviour changes, regardless of whether the signalling occurs directly through its own nuclear translocation or indirectly through its downstream target proteins [28,41]. In this study, interestingly, we found changes in the nuclear morphology of chondrocytes in response to the patterned equidistant micropillar substrates with different stiffnesses (Fig. 5). At the interface layer, although the nuclei displayed meshwork-like spreading on both the soft and stiff substrates, the spreading area of the nuclear region on the stiff substrate was larger than that on the soft substrate (especially for the cells marked with yellow circles, Fig. 5a, upper). To further confirm these changes in nuclear spreading areas, we counted the nuclear spreading area of 200 ​cells obtained from five independent experiments (Fig. 5b, source data) and confirmed that the nuclear spreading areas of chondrocytes on the stiff substrate were significantly higher than those on the soft substrate at the interface layer (Fig. 5b, a strong significant difference). At the interface above, we also observed a difference in the nuclear spreading areas of chondrocytes in response to micropillar substrates with different stiffnesses, which reached the threshold of a statistically significant difference (Fig. 5c, a weak significant difference with a p value of 0.0491). We sorted out all changed gene candidates that functioned in the nuclear region and clustered them (Fig. 5d). We also sorted out all changed transcription factors that had a direct interaction with gene activation and clustered them in Fig. 5e. The changes in nuclear spreading area might imply its correlation with nuclear activity and gene activation [42,43] and thus might partially influence the cell behaviour of chondrocytes.

### 3.5 Patterned equidistant stiff micropillars increase chondrocyte hypertrophy

**Fig. 6:**
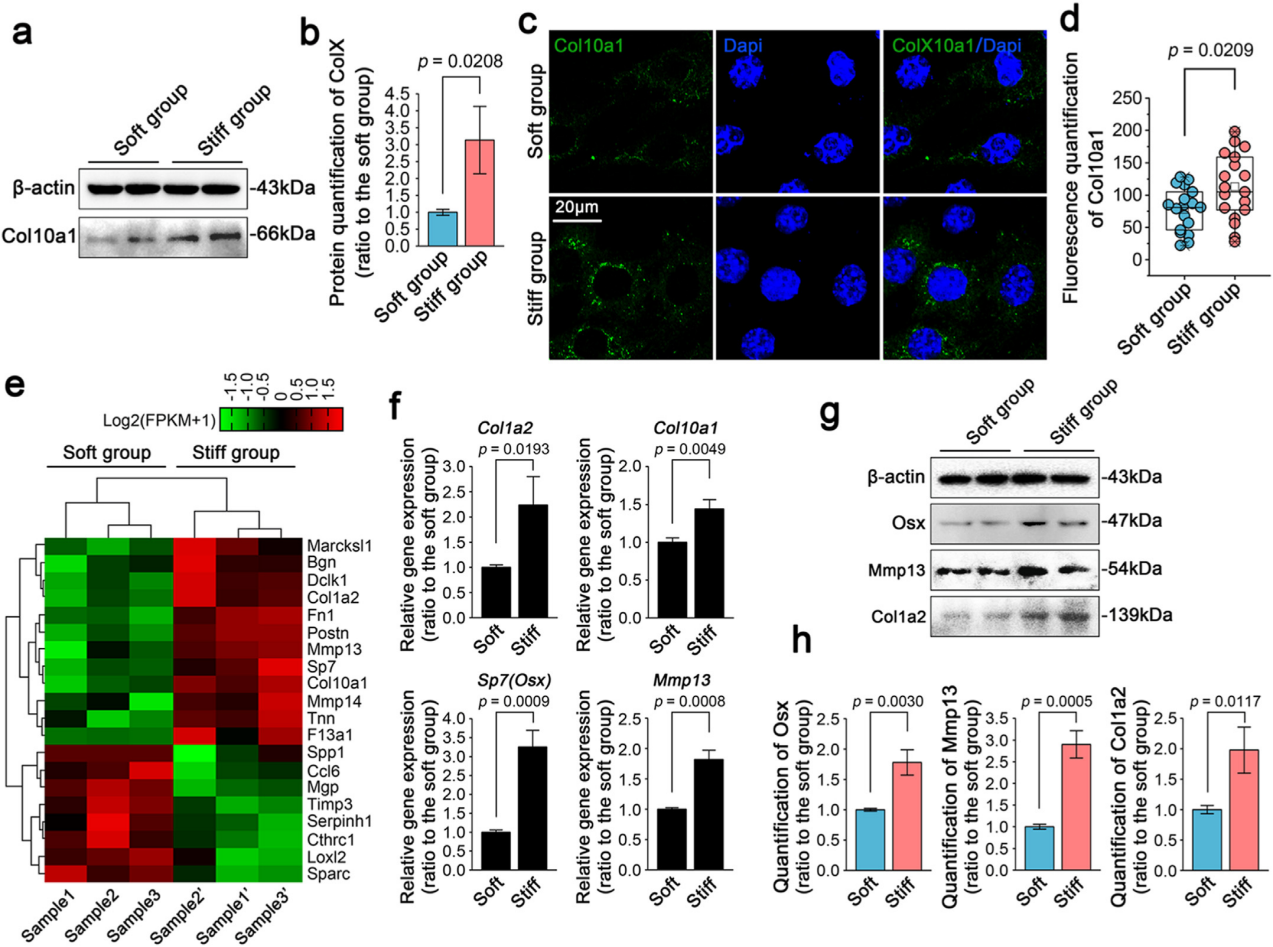
Patterned equidistant stiff micropillar substrate induced the hypertrophic differentiation of chondrocytes **a).** Western blotting indicating the changes of col10a1 in chondrocytes in response to patterned equidistant micropillar substrates with different stiffnesses. The images were based on three independent experiments (n ​= ​3). **b).** Quantification of Col10a1 in (a). The data were based on three independent experiments (n ​= ​3). **c).** Representative CLSM images indicating the changes of Col10a1 in chondrocytes in response to patterned equidistant micropillar substrates with different stiffnesses. The images were based on three independent experiments (n ​= ​3). **d).** Total fluorescent quantification of Col10a1 in (c). The data were based on 18 ​cells from three independent experiments. **e).** Pheatmap showing all changed genes related to chondrocyte hypertrophy in response to patterned equidistant micropillar substrates with different stiffnesses. The data originated from RNA sequencing. Samples 1 and 1′, 2 and 2′, and 3 and 3′, were from the same mother cells. The data were presented based on FPKM (Fragments per kilobase of exon model per million mapped fragments). **f).** qPCR showing the changes in typical gene markers of chondrocyte hypertrophy in response to patterned equidistant micropillar substrates with different stiffnesses. The data were based on three different independent experiments (n ​= ​3). **g).** Western blotting indicating the protein changes of these markers in chondrocytes in response to patterned equidistant micropillar substrates with different stiffnesses. The images were based on three independent experiments (n ​= ​3). **h).** Quantification of Osx, Mmp13 and Col1a2 in (g). The data were based on three independent experiments (n ​= ​3). The data presented in **d** are shown as box (from 25, 50–75%) and whisker (SD values) plots. The significant data presented in **b**, **d**, **f** and **h** are based on Two-tailed Student's t Tests (source data).

Most importantly, we showed the occurrence of chondrocyte hypertrophy in response to the patterned equidistant micropillar substrates with different stiffnesses. We first performed western blotting and found that the expression of Col10a1, a typical marker for chondrocyte hypertrophy, was greatly increased on the stiff substrate relative to that on the soft substrate (Fig. 6a). Quantitative analysis further confirmed the change in Col10a1 (Fig. 6b). We then used immunofluorescence and detected the intracellular expression of its pro-protein in chondrocytes (Fig. 6c). The results indicated that more chondrocytes expressed stronger Col10a1 on the stiff substrate than on the soft substrate. Quantitative analysis also showed an increase in Col10a1 in chondrocytes on the stiff substrate (Fig. 6d). More accurately, we then used RNA sequencing (Fig. 6e) and found that gene expression of Col10a1 was consistent with the results in its total protein content (Fig. 6a & b) and its cytoplasmic accumulation (Fig. 6c & d). In addition to Col10a1 (the most typical maker of chondrocyte hypertrophy), the changes of markers including Col1a2, Sp7 (Osx) and MMP13 were also detected by RNA sequencing (Fig. 6e). Col1a2 and MMP13 are well recognized to be the markers of chondrocyte hypertrophy [44,45], and Sp7 (Osx) is involved in the regulation of chondrocyte hypertrophy and its basal expression implies the transformation of mature chondrocytes towards hypertrophic chondrocytes [46]. Thus, the changes in these three markers also indicated the regulation of stiffness on hypertrophic chondrocytes. To further confirm the changes of these candidates at gene level, we performed qPCR (Fig. 6f) and the results showed that the expressions of Col1a2, Col10a1, Sp7 (Osx) and MMP13, were all up-regulated, which was consistent with the results of RNA sequencing in Fig. 6e. To further verify whether changes of Col1a2, MMP13 and Sp7 (Osx) at protein levels are consistent with gene expression, we performed western blotting (Fig. 6g) and the results showed the expressions of Col1a2, MMP13 and Sp7 (Osx) were all increased in chondrocytes on stiff micropillar substrates. The quantification of Col1a2, MMP13 and Sp7 (Osx) further confirmed these changes in protein level (Fig. 6h). Taken together, based on the biomimetic the collagen fibrillar network of the cartilage matrix by using patterned equidistant micropillar substrates, we verified the changes from cell morphologies, cytoskeleton-focal adhesion axis, Erk1/2/MAPK signalling, nuclear spreading areas to chondrocyte hypertrophy (Fig. 8).

### 3.6 Mouse ACLT-OA model validates the correlation between osteoid-like formation and cartilage hypertrophy

**Fig. 7:**
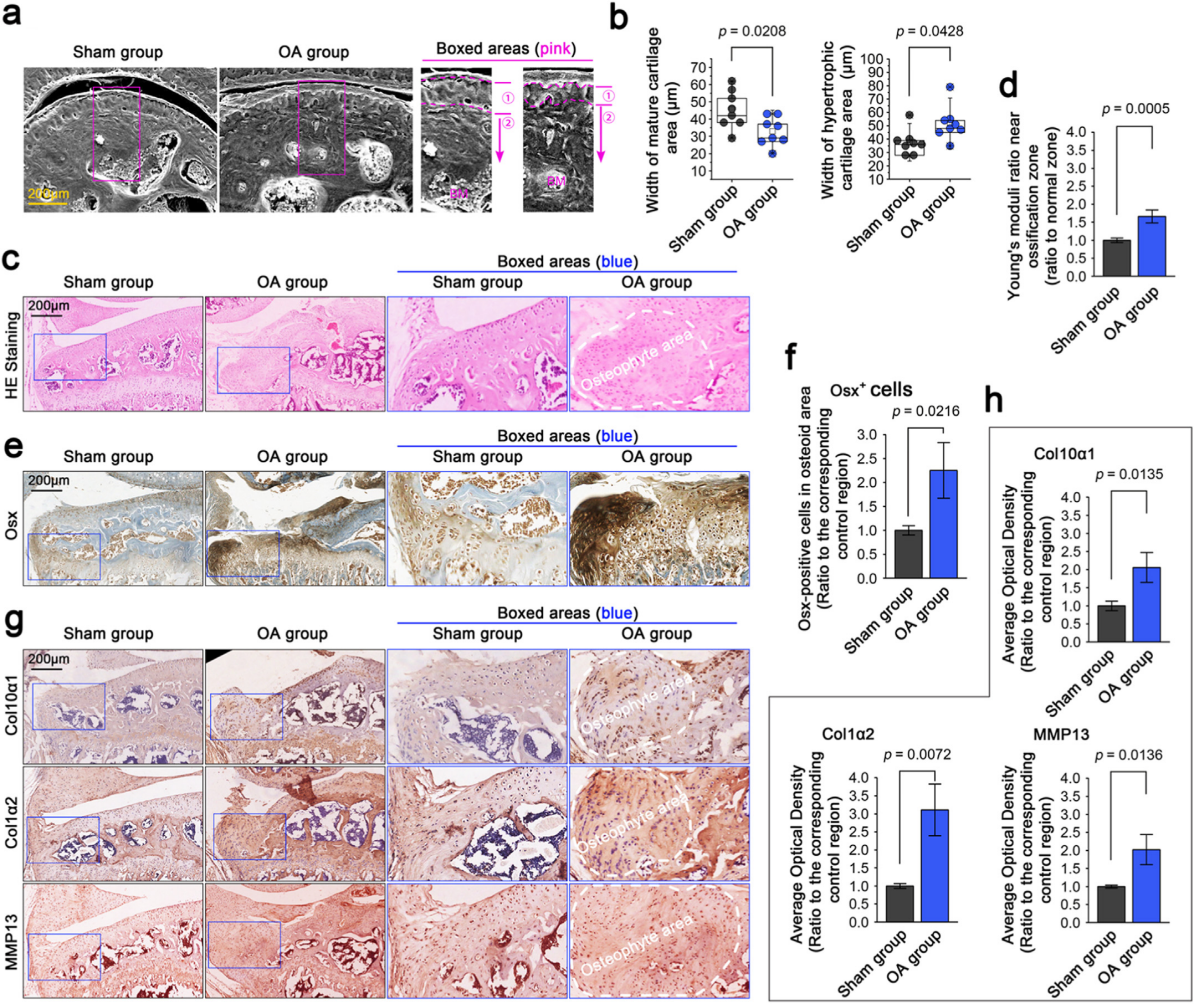
Changes in cartilage hypertrophy indicators in the ossified area of the knee joint in a mouse model of osteoarthritis. a). Representative SEM images indicating the changes in mature and hypertrophic cartilage areas after ACLT-OA modeling for 4 weeks. ① indicates the mature cartilage zone and ② indicates the hypertrophic cartilage zone. The images were chosen based on eight independent repeats (n ​= ​8). b). Quantification of the widths of mature and hypertrophic cartilage areas in the normal (sham) and OA groups. The data were analyzed based on eight independent repeats (n ​= ​8). c). HE staining showing the appearance of osteoid (osteophyte precursor) in the knee hypertrophic cartilage area of OA mice. The images were chosen based on four independent repeats (n ​= ​4). d). Nanoindentation showing the changes in Young's moduli at osteoid-like regions. The corresponding regions of the knee joint cartilage in normal (sham) mice serve as the control group. The data were analyzed based on four independent repeats (n ​= ​4). e). IHC staining showing the changes in Osx-positive cells at the site of osteoid in the knee hypertrophic cartilage area between the normal and OA groups. Osx, as a transcription factor, expresses in the nuclei of hypertrophic chondrocytes. The images were chosen based on three independent repeats (n ​= ​3). f). Quantification of Osx-positive cells at the site of osteoid in the knee hypertrophic cartilage area between the normal and OA groups. The data were analyzed based on three independent repeats (n ​= ​3). g). IHC staining showing the changes of Col10a1, Col1a2 and MMP13 at the site of osteoid in the knee hypertrophic cartilage area between the normal and OA groups. The images were chosen based on three independent repeats (n ​= ​3). h). Quantification of Col10a1, Col1a2 and MMP13 at the site of osteoid in the knee hypertrophic cartilage area between the normal and OA groups. The data were analyzed based on three independent repeats (n ​= ​3).

Finally, we established an OA mice model by using ACLT surgery. In the early stage of OA occurrence (4 weeks after ACLT), we observed changes in the cartilage layer by SEM: the mature cartilage layer (proliferating zone) had become thinner and hypertrophic cartilage layer (hypertrophic zone) had become thicker in OA mice than in normal (sham) ones (Fig. 7a). Quantification of the widths of these layers confirmed the results (Fig. 7b). In the later stages of OA (12 weeks after ACLT), we could observe that there were many osteophyte areas formed in the hypertrophic and calcified cartilage zone by HE staining (Fig. 7c). In these osteoid-like areas, we detected an increase in their stiffness by characterizing changes of Young's moduli through nanoindentation (Fig. 7d). Interestingly, we found more irregular hypertrophic chondrocytes generated in these osteoid-like areas, with an enhanced expression of Osx by IHC staining (Fig. 7e). These Osx positive chondrocytes are 2.25 times higher than normal hypertrophic chondrocytes at this location (Fig. 7f). Finally, we detected the hypertrophic markers including Col10a1, Col1a2 and MMP13 by IHC staining (Fig. 7g), and the results showed that these proteins were all increased in these osteoid-like areas in the OA group than the corresponding areas of the sham group. Quantification of these proteins further confirmed the results (Fig. 7h). Collectively, all the results about cartilage hypertrophy in the osteoid-like areas of OA mice model indicated their correlation and proved the conclusion of chondrocyte hypertrophy in response to stiffened equidistant micropillar substrate that mimicking the matrix stiffening in cartilage during the transition from a normal state to a state of osteoarthritis.

**Fig. 8:**
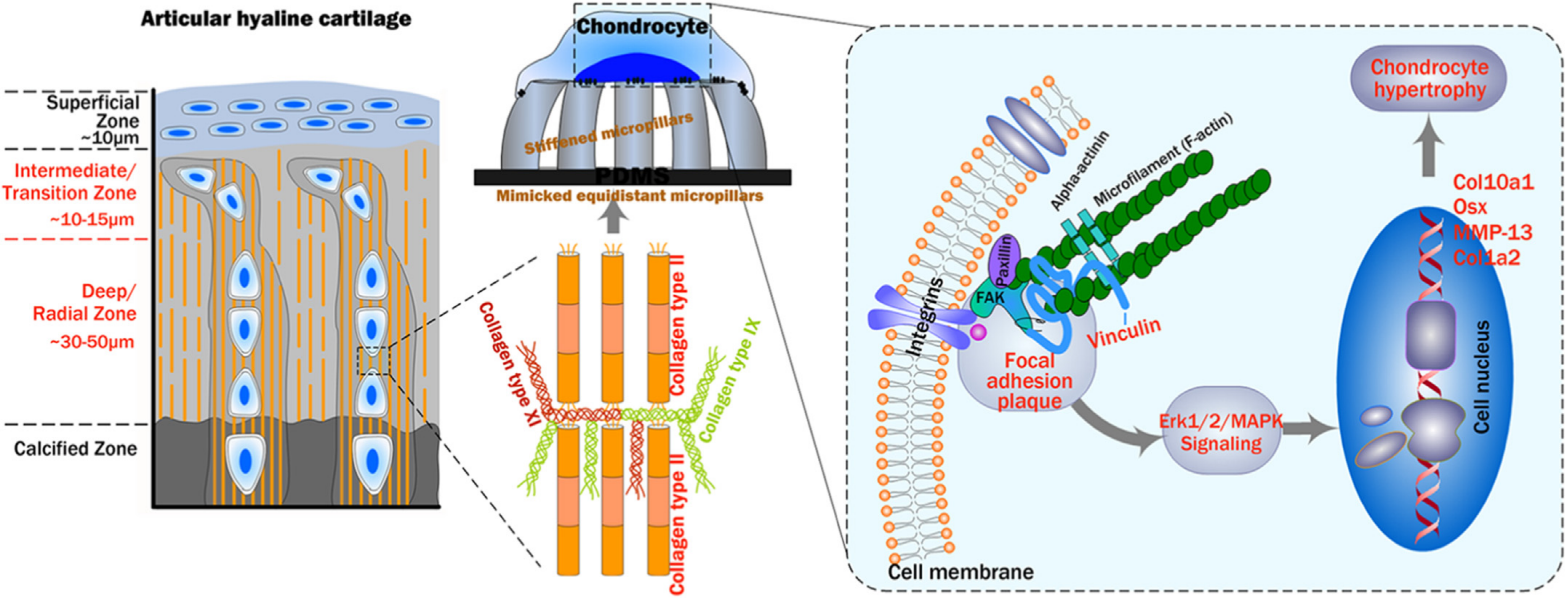
**The schematic diagram shows the hypertrophic response of chondrocytes in response to patterned equidistant micropillar substrates with different stiffnesses.** Chondrocytes undergo mechanical sensing, focal adhesion mechanotransduction (vinculin), and cytoplasmic signal activation (Erk1/2 signalling) to alter the protein markers including Col10a1, Col1a2, Osx and MMP13, and thus promote cartilage hypertrophy.

## 4 Discussion

Microenvironmental mechanical properties control cell functions and determine cell fate [47,48]. One of the most important determinants of microenvironmental mechanical properties is ECM stiffness, from adipose tissue with a low elastic modulus to bone tissue with a high elastic modulus [49]. The stiffness of the cartilage matrix surrounding chondrocytes is largely responsible for their shape transition from discoidal to spheroidal, their secretion efficiency of collagen and aggrecan and their phenotype maintenance [50]. In the current study, we designed a PDMS patterned equidistant micropillar to mimic the collagen fibrillar network of the cartilage matrix. On this basis, we changed the stiffness of these fibre-like micropillar substrates and investigated the cellular behaviour changes of chondrocytes in response to these soft/stiff micropillar substrates. The most notable finding in the current study was that the biomimicking stiffened fibre-like substrate facilitates chondrocyte hypertrophy after changing a series of subcellular components, the including cytoskeleton, focal adhesion plaque, Erk/MAPK signalling and nuclear spreading area. These results are beneficial for interpreting the cell function changes of chondrocytes affected by tissue stiffening during ageing and OA disease.

As typical mechanosensitive cells, chondrocytes are able to sense external mechanical changes through exerting contraction forces on the surrounding microenvironment and consequently adjust their endogenous cytoskeleton contractility to achieve cytoskeletal rearrangement [51] and mediate their cell-ECM adhesion strength by changing the insoluble focal adhesion composition and size [52,53]. From previous data based on two-dimensional (2D) culture, we know that the spreading area of cells becomes larger when cells are seeded onto stiffened substrates; moreover, cells produce more orderly microfilament bundles on stiffened substrates [24,30,32]. The chondrocyte cytoskeleton consists of three important components, i.e., actin microfilament, microtubules and vimentin intermediate filaments [54]. Among these three components, actin microfilaments are recognized to be the most sensitive to mechanical stresses. For example, stiffnesses based on 2D culture mediate actin filament distribution and influence cellular synapse generation [24]. The cortical actin depolymerization of chondrocytes is enhanced by compressive strain [55]. Mechanical loadings such as cyclic tensile strain [56], compressive strain [57] and hydrostatic pressure [58] induce the rearrangement of actin stress fibres of chondrocytes; Moreover, the enhancement of actin stress fibres by cyclic tensile strain facilitates the activation of Rho A, a member of the Rho GTPase family, and triggers intracellular Rho-ROCK signalling to regulate actomyosin contractility and cell shape [59]. In our current study, based on mimicked fibre-like equidistant micropillar substrates, we found that more actin filament bundles of chondrocytes were formed in response to stiff micropillar substrates (Fig. 2c), and the later formation of different types of microfilament bundles might further determine the fate of chondrocyte hypertrophy (Fig. 2, Fig. 6c).

To counterbalance the endogenous cytoskeleton contractility, cells adjust their cell-ECM adhesion strength by changing the focal adhesion plaque to regulate cellular activity. Previous reports showed that a stiffened substrate based on 2D culture enhanced the expression of focal adhesion kinase (FAK) by forming a larger size in the whole cytoplasmic region [24] and increased the plaque-like formation of paxillin [27] and vinculin [23] at the boundary of the cell membrane. Cyclic hydrostatic compression impacts FAK-based signal transduction and regulates the apoptosis of meniscal cartilage [60]. Periodic mechanical stress promotes FAK phosphorylation to induce chondrocyte proliferation [61]. Cyclic tensile strain activated the phosphorylation of FAK and upregulated proinflammatory cytokine expression [62]. Hydrostatic compression increased the expression of FAK and enhanced the viability of chondrocytes [63]. In this study, we screened out the changes, including integrins (Itgax), Ptk2 (FAK), paxillin (Pxn) and vinculin (Vcl), at the transcriptional level (Fig. 2b), and characterized the expression and distribution of vinculin in chondrocytes in response to these fibre-like equidistant micropillar substrates with different stiffnesses (Fig. 3). We provided more reliable evidence to show that the rearrangement of the cytoskeleton and the change in focal adhesion plaque in chondrocytes in response to different stiffnesses because these results were based on the biomimicking physical microenvironment that was much closer to that of the ECM of real cartilage in vivo.

The interaction between chondrocytes and microenvironment stiffness mainly involves two adhesion complexes: adherens junctions (AJs) and focal adhesions (FAs) [64]. AJs are responsible for cell-cell interactions, while FAs mainly achieve cell-matrix interactions. The changes in FAs, such as vinculin (Fig. 3), in chondrocytes, trigger the cell responses that are associated with cytoskeletal reorganization, leading to changes in cell shape (Fig. 1d–g), actin microfilament bundling and reorganization (Fig. 2c–e), and the initiation of mechanotransduction of physical signalling into intracellular biochemical signalling [39,40]. There are many intracellular signal pathways that can be activated by microenvironment mechanics. They include but are not limited to integrins, Rho/Rock, TGF/BMPs, FGFs, protein kinase C (PKC), phosphoinositide 3-kinase (PI3K)–protein kinase B (AKT), Hippo/Yap, wnt/β-catenin and MAPK [65]. In the current study, chondrocytes perceived the stiffness changes of patterned equidistant fibre-like micropillars and mainly triggered four kinds of intracellular signalling (Fig. 4a). Among them, the factors regulating the changes in MAPK signalling were the most abundant. MAPK is a well-recognized intracellular signalling pathway and has been implicated in many aspects of cartilage biology, such as endochondral ossification [66] and cartilage matrix synthesis and remodelling [67]. It can also play a vital role in the conversion of a series of extracellular stimuli into cellular responses, such as cell viability, migration, proliferation and differentiation [68]. Here, we found that stiffened equidistant fibre-like micropillars activated Erk1/2/MAPK signalling, and induced chondrocyte hypertrophy. Interestingly, a hallmark of the pathogenesis and progression of OA is the change in ECM stiffness [[14], [15], [16], [17], [18]], which is associated with loss of proteoglycan, accumulation of ECM collagen types, unbundling of prototypic collagen fibrils and enhancement of dissimilatory cross-linking of the collagen network with proteoglycan depletion [14,16]. Moreover, the proliferation zone of OA cartilage, where mature chondrocytes reside, becomes thinner, and the hypertrophic area of OA cartilage becomes thicker, so that the ability of cartilage to resist loads is gradually lost [3,9]. With the process of pathological ECM stiffening, there were some cues referring the relationship between changes in signalling pathways and hypertrophy of chondrocytes. For example, Tong et al. found that Wnt16 inhibited chondrocyte hypertrophy via a PCP/JNK-mTORC1-PTHrP cascade, which in turn prevented the progression of OA [69]. The result about wnt signalling was consistent with our gene ontology (GO) analysis of wnts (Fig. 4a). Regarding ERK1/2 signalling, there have been no reports indicating that the *p*-ERK1/2 pathway is involved in the regulation of chondrocyte hypertrophy, but our current results confirm this. Interestingly, some studies have shown that the ERK1/2 signalling is involved in the regulation of cardiomyocyte hypertrophy and is considered to be the most important signal leading to maladaptive cardiac hypertrophy [70,71]. These results in myocardial hypertrophy provide some indirect evidence for our conclusion in the current research. Collectively, as the activation of MAPK signalling was implicated in the catabolic metabolism and matrix remodelling of chondrocytes [72], it can be inferred that the result about stiffened matrix-mediated chondrocyte hypertrophy through Erk/MAPK signalling might be of great importance to the changes in matrix structure and tissue metabolism.

Another important issue in this study was the change in the nuclear spreading areas at the interface between chondrocytes and the top surface of micropillars (Fig. 5a–c). In other words, at this interface layer, the nuclear spreading area might be reshaped. Reshaping the nuclear area is noteworthy because it can potentially involve gene regulation [43,73]. However, reshaping the nuclear area is not easy to achieve due to nuclear physical properties such as nuclear compliance, resistance to deformation and requirements for steady active forces generated by actomyosin contraction, actin microfilament or microtubule polymerization, and microtubule motor capacity to compress or pull on the nuclei [43]. To date, recognized evidence has shown that the linker of the nucleoskeleton and cytoskeleton complex (LINC), which can transmit mechanical stress from the cytoskeleton to the nucleus, has a great impact on nuclear activities, including nucleolar morphology, chromatin remodelling, histone distribution and transcriptional activity [73], thus partially influencing the majority of cell behaviour. In this study, we detected a change in the nuclear spreading area of chondrocytes at the interface layer, and this change might have a close correlation with nuclear activity and gene activation because nuclear mediators (Fig. 5d) and transcription factors (Fig. 5e) were detected, although it is not ruled out that these changes in nuclear mediators and transcription factors were the result of other cytoplasmic signals.

Currently, many new strategies for OA therapy have emerged based on novel bionanomaterials, such as engineered scaffolds for nano-delivered drugs, nanoprobes and nanorobots [74,75]. For instance, Zhang et al. showed that injectable meloxicam-loaded nano-liposomes into the temporomandibular joint cavity for OA treatment had both anti-inflammatory and lubricating effects, and had achieved good curative effect in animal disease models [76]. Nativel et al. showed that injection of mesenchymal stem cells encapsulated in nano-hydrogels into the joint cavity was also effective in the treatment of arthritis [77]. All these results suggest that drug-loaded scaffolds made from nanomimetic materials have great promise for the treatment of osteoarticular-related diseases. However, in order to achieve better therapeutic effects on OA, we often need to have a deeper understanding of the pathogenesis of OA, changes in OA joint composition, and pathological manifestations of OA cartilage. In our current study, this novel PDMS-based patterned micropillar substrate we designed has a similar topology to the collagen fiber network of cartilage matrix, which enables us to dissect the real process of OA onset and deterioration. The understanding about the internal biological changes of OA might bring more potent and effective therapy for OA disease based on deep biomimetic cartilage engineering. Facing the clinical challenges such as potential toxicity, lack of biodegradability that may lead to chronic inflammation and other accompanying complications and problems, designing advanced, biodegradable materials that can fully mimic the extracellular matrix fiber framework of cartilage wound potentially promote the clinical treatment of cartilage defects and OA cartilage lesions.

With the development of materials science and clinical medicine, more and more scholars began to try whether bionic artificial materials can be served as a replacement for diseased tissues or organs. In recent years, breakthroughs have been made in the research field of bionic medicine, such as the inventions of various hemostatic materials, artificial hearts, implants, artificial periosteum and bone power [[78], [79], [80]]. Interestingly, PDMS are widely used in the biomedical field for their good biocompatibility, optical transparency, and easy handing [81]. As early as more than ten years ago, Tanaka et al. made cardiomyocyte bio-microdrives using PDMS micropillars [82]. More recently, Potrich et al. utilized the plasticity property of PDMS for biomedical studies of microfluidic devices for biological applications [83]. All these results indicate that PDMS has great promise for applications in clinical medicine. It is well known that articular cartilage is difficult to repair after damage due to its unique biological properties (avascular, nerve-free and lymphatic-free). Interestingly, we designed a novel PDMS-based patterned isometric micropillar substrate to mimic the collagen fiber network of cartilage matrix. Although the bionic fiber-like isometric micropillar substrates cannot fully represent the vivo 3D environment of articular cartilage in the current study, they provide a new way to simulate the collagen fiber network of cartilage matrix. As we continue to improve and optimize the parameters of PDMS-patterned isometric micropillars, such as shortening the diameter of the micropillars, reducing the space among the micropillars and changing the orientation of the micropillars, we would established a collagen fiber bionic material that more closely resembles the real microenvironment. This advanced biomimetic material would be more conducive to the treatment of joint-related diseases.

We acknowledge some limitations in the current study. First, we know that, as chondrocytes live in a three-dimensional (3D) environment in vivo, their phenotypes are dependent on complicated but elaborate cell-cell and cell-matrix interactions [65]. In 2D cell cultures, cell-cell and cell-matrix interactions are greatly limited and impaired. In the current study, the biomimicking fibril-like equidistant micropillar substrates cannot fully represent a 3D environment; thus, the study only provides data that are much closer to the real response involving matrix stiffness. Second, we aim to establish a biomimicking collagen fibril-like physical microenvironment based on these PDMS-patterned equidistant micropillars. The diameter of the patterned equidistant micropillars used in this study is 5 ​μm. However, the densely packed collagen fibrils around chondrocytes in vivo are arranged radially, and some of them are straight at 30 ​nm, while others are in an opposed spiral arrangement below 10 ​nm. Moreover, the diameter of these packed collagen fibrils is below 200 ​nm, and the pore size of the collagen fibril network is 60–200 ​nm [73], which is far smaller than the diameter used in this study. This might lead to the fact that the data in this study only reflect changes in cell behaviour in a general view. More work is needed to improve the feasibility of generating fine fibril-like micropillars or advanced biomaterials closer to the real microenvironment. Furthermore, in this study we focused on designing a microenvironment that can actually mimic the collagen network of the articular cartilage matrix and investigating the morphological changes of chondrocytes at different substrate stiffnesses. Our results above suggest that stiff micropillar may induce chondrocyte hypertrophy, which is consistent with the stiffening of the collagen matrix and the hypertrophy of chondrocyte during the progression of OA in vivo. This provides us with a new bionic material to investigate the pathogenesis of OA and to treat osteoarthritic disease. However, due to its many drawbacks mentioned above, it has not yet entered in vivo studies. Interestingly, in recent years, many researchers have started to explore experiments in vivo by using PDMS as a bionic material as a medium [84]. For instance, Kim et al. found that bionic materials made with PDMS had better affinity for cerebrospinal fluid and minimal damaged to neuronal tissues with less immune reactions, which could be more beneficial for research work related to brain function [85]. These studies show that PDMS substrates have also great potential for application in both in vivo and *in vitro* experimental studies. Collectively, with the continuous breakthroughs on our technology, PDMS micropillar substrates might play an important role in the field of articular cartilage disease model research.

## Credit author statement

Mengmeng Duan: Methodology, Software, Formal analysis, Investigation, Writing – original draft; Shuang Xia: Methodology & Data curation; Yang Liu: Investigation & Methodology; Xiaohua Pu: Investigation & Methodology; Yukun Chen: Methodology & Data curation; Yilin Zhou: Methodology & Data curation; Minglei Huang: Investigation & Methodology; Demao Zhang: Data curation, Formal analysis, Validation & Supervision; Caixia Pi: Formal analysis, Validation & Supervision; Jing Xie: Conceptualization, Methodology, Resources, Writing – review & editing, Supervision, Project administration & Funding acquisition

## Declaration of competing interest

The authors declare that they have no known competing financial interests or personal relationships that could have appeared to influence the work reported in this paper.

## Data Availability

Data will be made available on request.
