# Supplementary material for: Stiffened fibre-like microenvironment based on patterned equidistant micropillars directs chondrocyte hypertrophy
Source: Mater Today Bio. 2023 May 27;20:100682. doi: 10.1016/j.mtbio.2023.100682 (PMC10251154; doi:10.1016/j.mtbio.2023.100682)
Supplement: Multimedia component 1 [file mmc1.doc]

Supplementary material for original article

**Stiffened fibre-like microenvironment based on patterned equidistant micropillars directs chondrocyte hypertrophy**

Mengmeng Duan, Shuang Xia, Yang Liu, Xiaohua Pu, Yukun Chen, Yilin Zhou, Minglei Huang, Caixia Pi, Demao Zhang, Jing Xie

**Supplementary figures**

**Figure S1**


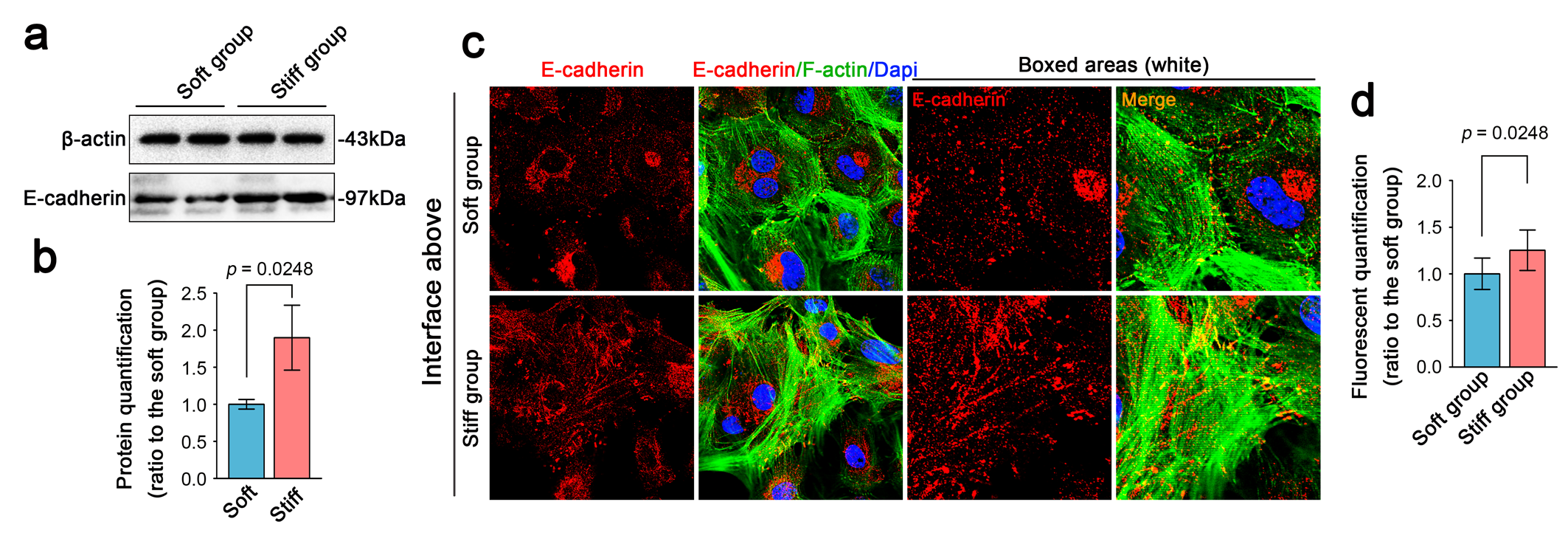


**Figure S1. The expression changes of E-cadherin in chondrocytes in response to PDMS-patterned equidistant micropillar substrates with different stiffnesses.**

a). Western blotting showing the protein changes of E-cadherin in chondrocytes in response to the patterned equidistant micropillar substrates with different stiffnesses. The results were based on three independent experiments (n = 3).

b). Quantification of E-cadherin in (a). The data were based on three different independent experiments (n = 3).

c). Representative CLSM images indicating the distribution changes of E-cadherin between adjacent chondrocytes in response to the patterned equidistant micropillar substrates with different stiffnesses. These images were obtained from the interface above and were chosen based on three independent experiments (n = 3).

d). Quantification of total fluorescent optical density in E-cadherin per chondrocytes in response to patterned equidistant micropillar substrates with different stiffnesses. The analysis was based on eight cells from three independent experiments (n = 3).
